# T-Cell Prolymphocytic Leukemia: Diagnosis, Pathogenesis, and Treatment

**DOI:** 10.3390/ijms241512106

**Published:** 2023-07-28

**Authors:** Marc Gutierrez, Patrick Bladek, Busra Goksu, Carlos Murga-Zamalloa, Dale Bixby, Ryan Wilcox

**Affiliations:** 1 Department of Internal Medicine, University of Michigan, Ann Arbor, MI 48109, USA; redstrat@med.umich.edu; 2 Department of Pathology, University of Illinois Chicago, Chicago, IL 60607, USA; pblade2@uic.edu (P.B.); bbacik2@uic.edu (B.G.); catto@uic.edu (C.M.-Z.); 3 Department of Internal Medicine, Division of Hematology and Oncology, University of Michigan, Ann Arbor, MI 60607, USA; dbixby@med.umich.edu

**Keywords:** T-prolymphocytic leukemia (T-PLL), TCL1A, ATM, alemtuzumab, T-cell receptor, JAK, STAT, BH3 mimetic

## Abstract

T-cell prolymphocytic leukemia (T-PLL) is a rare and aggressive neoplasm of mature T-cells. Most patients with T-PLL present with lymphocytosis, anemia, thrombocytopenia, and hepatosplenomegaly. Correct identification of T-PLL is essential because treatment for this disease is distinct from that of other T-cell neoplasms. In 2019, the T-PLL International Study Group (TPLL-ISG) established criteria for the diagnosis, staging, and assessment of response to treatment of T-PLL with the goal of harmonizing research efforts and supporting clinical decision-making. T-PLL pathogenesis is commonly driven by T-cell leukemia 1 (*TCL1*) overexpression and *ATM* loss, genetic alterations that are incorporated into the TPLL-ISG diagnostic criteria. The cooperativity between *TCL1* family members and *ATM* is seemingly unique to T-PLL across the spectrum of T-cell neoplasms. The role of the T-cell receptor, its downstream kinases, and JAK/STAT signaling are also emerging themes in disease pathogenesis and have obvious therapeutic implications. Despite improved understanding of disease pathogenesis, alemtuzumab remains the frontline therapy in the treatment of naïve patients with indications for treatment given its high response rate. Unfortunately, the responses achieved are rarely durable, and the majority of patients are not candidates for consolidation with hematopoietic stem cell transplantation. Improved understanding of T-PLL pathogenesis has unveiled novel therapeutic vulnerabilities that may change the natural history of this lymphoproliferative neoplasm and will be the focus of this concise review.

## 1. Introduction

T-cell prolymphocytic leukemia (T-PLL), distinct from chronic lymphocytic leukemia (CLL) and B-cell prolymphocytic processes, was first described by Catovsky and colleagues in 1973 [1]. However, the moniker (“prolymphocytic”) remains a misnomer, as the leukemic T-cells are post-thymic in origin. T-PLL is rare, with an incidence of only 2 per million per year in Western countries, accounting for about 2% of mature leukemias [2]. The most recent update in the WHO classification incorporated diagnostic recommendations from the T-PLL International Study Group (TPLL-ISG) assembled in 2019 [3]. The latest update of the International Consensus Classification (ICC) of Mature Lymphoid Neoplasms has not changed the definition of T-PLL [4,5]. Prior to the use of the anti-CD52 monoclonal antibody alemtuzumab and allogeneic stem cell transplantation, T-PLL was incurable and median overall survival was measured in months [6], highlighting the importance of clinical recognition and appropriate disease classification. As salvage therapies after relapse or progression in alemtuzumab-treated patients are suboptimal, the median overall survival from the time of diagnosis is less than 3 years in the alemtuzumab era [6].

## 2. Clinical Presentation

Since the initial description of T-PLL, reports of its presenting characteristics are largely confined to a limited number of small case series [6,7,8,9]. Most patients afflicted with T-PLL present with extreme lymphocytosis (≥100,000/µL), anemia, thrombocytopenia, hepatosplenomegaly, and non-bulky lymphadenopathy in the 6th to 7th decade of life [3]. Individuals with ataxia telangiectasia due to loss of the tumor suppressor gene ataxia telangiectasia mutated (ATM) present during adolescence [10]. Extranodal, including cutaneous, involvement may be observed, while central nervous system involvement is rare. Peripheral edema, including periorbital and conjunctival edema, and serous (pleural or peritoneal) effusions may occur in up to one-quarter of patients. While most patients present with widely disseminated and rapidly progressive disease, a minority of patients present with asymptomatic (“inactive”) disease, which may slowly progress over the years [9]. The average time between diagnosis and treatment initiation is ≈2 months, but is highly variable (range: <1 month to ≈4 years) given the minority of patients with asymptomatic and indolent disease [9].

## 3. Diagnosis

**Figure 1:**
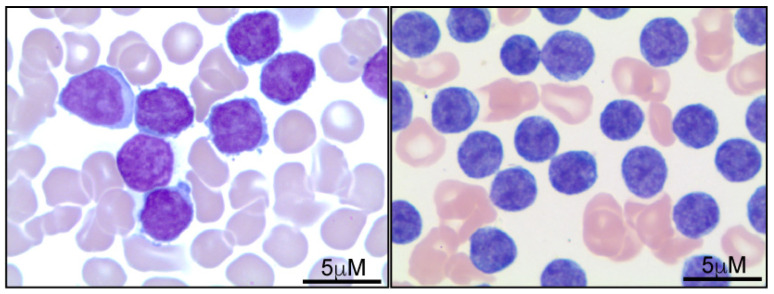
Peripheral blood involvement by T-PLL. Representative images demonstrate that T-PLL tumor cells (Wright stain) can be variable in size and range from medium (**right panel**) to large (**left panel**), with irregular nuclear contours, condensed chromatin, and prominent nucleoli. The morphological and cytological features of T-PLL have been previously documented and described [2,6,7].

Establishing a diagnosis of T-PLL requires confirmation of neoplastic involvement in the peripheral blood or bone marrow. Histologically, three morphologic variants have been recognized; the most common (50–75%) predominantly comprises medium-sized lymphocytes with a high nuclear-to-cytoplasmic ratio, condensed chromatin, and single prominent nucleoli (Figure 1). The second morphologic variant (20–25%) is characterized by atypical lymphocytes with convoluted nuclear contours that resemble the cells seen in Sézary Syndrome. The third morphological variant features heterogeneous populations of atypical lymphocytes that comprise small lymphocytes that are similar to those identified in chronic lymphocytic leukemia (CLL) and, to a lesser extent, medium-sized forms that are similar to those identified in the most common morphological pattern of T-PLL [1,3,6,7,11,12,13]. 

T-PLL cells have an immunophenotype consistent with their derivation from mature (post-thymic) T-cells, including absent expression of TdT and CD1a. Most cases express CD3, although the surface expression of CD3 may be decreased or absent in some cases. The expression of CD4 without CD8 is the most frequent immunophenotype (58–65%). Less commonly (21–32%), tumor cells co-express CD4 and CD8. The aberrant loss of the pan-T-cell markers CD2, CD5, and CD7 is not commonly observed [6]. CD52 expression is almost uniformly observed in untreated patients and is of obvious therapeutic relevance [14]. T-cell receptor, usually αβ, expression is typically retained and is consistent with both its increasingly appreciated role in disease pathogenesis (see below) and a broader “three signal model” of disease pathogenesis [15].

In addition to T-PLL, alternative T-cell lymphoproliferative neoplasms derived from post-thymic T-cells may present with leukemic involvement (Table 1) [16]. Acute T-cell leukemia/lymphoma (ATLL), which is associated with HTLV-1 infection, features leukemic involvement by neoplastic lymphocytes with variable morphology, ranging from small to large neoplastic lymphocytes, occasionally including “flower-like” forms. Immunophenotypically, ATLL frequently shows CD4-positive T-cells with co-expression of CD25 and FoxP3 with lack of CD7 expression. Importantly, detection of HTLV-1 infection in the neoplastic compartment by serology or molecular testing is required to establish an ATLL diagnosis [16]. T-cell large granular lymphocytic leukemia (T-LGL) is characterized by absolute lymphocytosis, with an expansion of T-cell large granular lymphocytes with no definitive morphological atypia. An indolent clinical course characterizes T-LGL; patients usually present with absolute neutropenia or anemia. Like T-LGL, NK-cell LGL are clinically indolent, but are characterized by an expansion of CD94-positive NK cells. In contrast, aggressive NK-cell leukemia (ANKL) is a rare disorder characterized by a fulminant clinical course with a median overall survival of 2 months. In addition to leukemic involvement, CNS and skin involvement may also be present in ANKL [17]; in contrast to T-PLL, the neoplastic cells in ANKL feature absent expression of surface CD3 and frequently co-express CD56 and CD94 antigens. 

**Figure 2:**
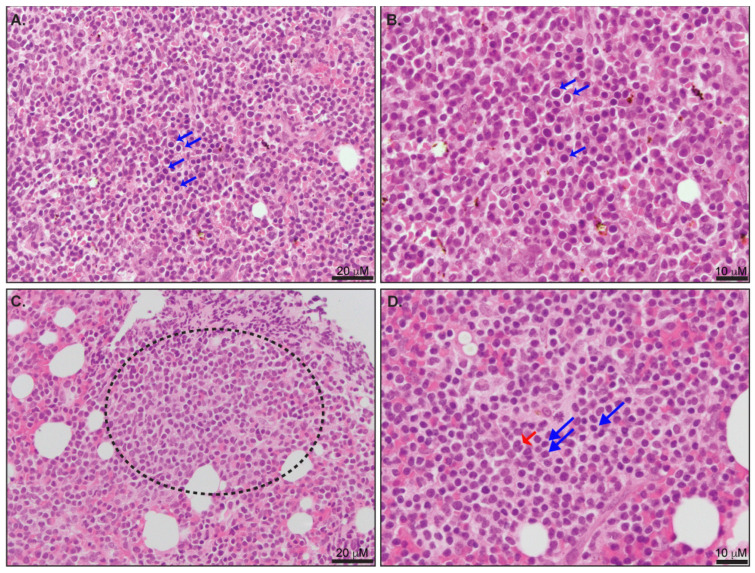
(**A**,**B**) Involvement of bone marrow with T-PLL. (**A**) Highlights of interstitial involvement by tumor cells (blue arrows). (**B**) The most common morphological pattern is composed of medium lymphocytes with round nuclear contours and central prominent nucleoli (blue arrows). (**C**) Focal aggregates of tumor cells are usually observed (dotted circles). (**D**) Small-cell morphology variant is characterized by small to medium forms with condensed chromatin (blue arrows). Occasional large “cerebriform” lymphocytes are also observed (red arrows). The morphological features characteristic of T-PLL bone marrow involvement have been previously described [2,6,7].

A bone marrow biopsy is not required if the diagnosis has been established in the peripheral blood. The tumor cells predominantly involve the bone marrow in an interstitial pattern, intermixed with background hematopoietic elements and focal small nodular aggregates (Figure 2). This pattern of infiltration may resemble other T-cell lymphoproliferative disorders, including ATLL and T-LGL (Table 1). During the bone marrow biopsy evaluation of T-PLL at therapy follow-up, a diagnostic pitfall is the normal positive expression of TCL1 by resident bone marrow hematogones [29]. Therefore, a combination of TCL1 expression and documentation of the expansion of clonal T-cells by immunohistochemistry and flow cytometry analysis is required to establish bone marrow involvement [29]. 

**Figure 3:**
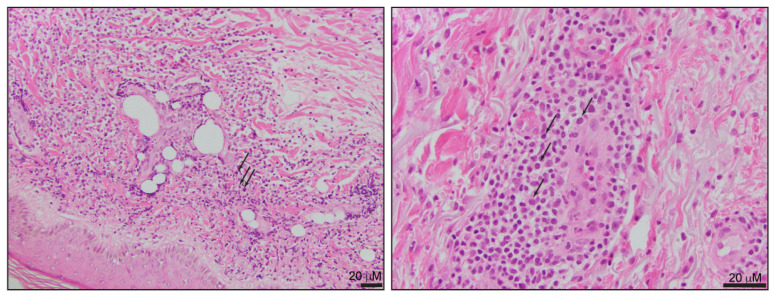
Skin involvement by T-PLL (black arrows). The tumor cells are predominantly distributed in a perivascular fashion, with no epidermotropism. Characteristic histological features of dermal involvement by T-PLL have been previously described [2,6,7].

The skin is the most common extramedullary site of T-PLL involvement and is more frequently observed in patients with a greater leukemic disease burden [30,31]. Skin involvement can be the initial presenting feature of T-PLL, but this is always accompanied by leukemic involvement, and concurrent evaluation of the peripheral blood must be performed in patients with this presentation [30,31]. Skin lesions include diffuse erythematous papules, macules, and plaques, sometimes in combination with ulcerations. A single-center retrospective study that included 41 T-PLL patients indicated that the skin is the most commonly biopsied site, even in patients with other extramedullary infiltrates, such as the liver or spleen [32]. The dermal infiltrate is characterized by small- to medium-size lymphocytes with predominant perivascular distribution, absent epidermotropism, and inconspicuous cytological atypia (Figure 3). The morphological features may be challenging to differentiate from other cutaneous T-cell neoplasms, including Sézary syndrome. 

Other extramedullary sites of involvement include the spleen, liver, and lymph nodes. According to the disease stage, liver involvement is characterized by varying degrees of periportal infiltrates and portal tract expansion [30]. Other locations include the bronchi, which are commonly associated with pleural effusions. Lymph node involvement is characterized by effacement of the nodal architecture by populations of small- to medium-size lymphocytes with minimal cytological atypia. Aleukemic presentations of T-PLL are extremely rare, with only two cases reported in the literature featuring white blood cell counts within normal limits, even after 26 months of follow-up [33]. The two cases reported bone marrow involvement and extensive lymphadenopathy with biopsy-proven disease involvement and TCL1 rearrangements detected by FISH. 

As noted, T-PLL is rare and associated with a divergent natural history—ranging from a rapidly progressive disease to one associated with prolonged periods of disease “inactivity”—frustrating efforts to compare case reports, retrospective studies, and small trials from different centers using different diagnostic criteria and therapeutic strategies. In 2019, the T-PLL International Study Group (TPLL-ISG) developed major and minor diagnostic criteria, as described in Table 2 [3]. The diagnosis is established if three major criteria or the first two major criteria and at least one minor criterion are satisfied. The constellation of clinical, histopathologic, serologic (e.g., HTLV-1 status), and genetic characteristics associated with T-PLL are usually sufficient to permit its discrimination from other T-cell-derived leukemias (e.g., T-cell large granular lymphocytic leukemia, adult T-cell leukemia/lymphoma, Sézary Syndrome) (Table 1). 

According to the proposed criteria, the cases that are negative for TCL1A, TCL1B, or MTCP1 rearrangements with characteristic clinical features of T-PLL should be considered TCL1-family negative T-PLL [3]. However, the distinction between these cases from peripheral T-cell lymphoma, not otherwise specified (PTCL, NOS), with leukemic involvement is complex. The largest series of T-cell lymphomas with a leukemic presentation and negative TCL1-family rearrangements included 16 PTCL, NOS patients [34]. In this series, the clinical features, immunophenotype, and overall survival were not different between PTCL, NOS cases with leukemic involvement (TCL1-family-negative), or T-PLL (TCL1-family-positive) cases. However, lymphadenopathy was more extensive in TCL1-family-negative cases [34]. Clinical presentations with an absolute lymphocyte count >5 × 10^9^/L, a clinical presentation characteristic of T-PLL, including characteristic skin lesions, splenomegaly, or effusions, and characteristic chromosomal abnormalities (Table 2), favor a diagnosis of TCL1-family negative T-PLL over PTCL, NOS with leukemic involvement. 

## 4. Risk Stratification

Clinical signs and symptoms seemingly do not correlate with prognosis in T-PLL, with the exception of pleural effusions, which have been associated with an increased risk of death [9]. While skin involvement is associated with significantly higher tumor cell nuclear factor-κB (NF-κB) expression, it has not been associated with reduced overall survival [31].

The largest retrospective T-PLL study to date included 119 patients, including 75 untreated and 43 previously treated patients. The median age at diagnosis was 63. During the period of review (1990 to 2016), 80% of patients died. On univariate analysis, factors associated with worse median overall survival included the presence of pleural effusion (10.8 months vs. 22.3 months), presence of TCL-1 by cytogenetics (16 months vs. 26 months), leukocytosis ≥ 208 K/µL (11.1 months vs. 22.3 months), anemia (8.4 months vs. 18.7 months), LDH ≥ 1668 IU/L (13.1 months vs. 26.5 months), and non-Caucasian identity (12 months vs. 21.9 months). Factors associated with decreased median progression-free survival on univariate analysis included anemia (1.3 months vs. 9.2 months), leukocytosis ≥ 208 K/µL (3.4 months vs. 9.2 months), non-Caucasian identity (3.9 months vs. 9.2 months), and patients on a treatment regimen not including alemtuzumab (3 months vs. 9.2 months). On multivariate analysis, factors that independently predicted death included anemia (hazard ratio (HR) 2.6) and elevated LDH (HR 2.3). Elevated beta-2-microglobulin ≥ 8 mg/L (HR 3.5), absence of small cell variant morphology (HR 3.7), and non-Caucasian identity (HR 1.7) independently predicted progression on multivariate analysis [9].

Increased TCL1 expression, T-cell receptor expression, and AKT activation have been associated with shorter lymphocyte doubling time and poorer prognosis [35]. Complex cytogenetics (e.g., >3 chromosomal abnormalities) have been associated with poorer overall survival [36]. With the possible exception of patients presenting with asymptomatic (“inactive”) T-PLL, risk stratification in T-PLL seemingly distinguishes those with poor survival (e.g., <2 years) from those with poorer (e.g., <12 months) survival, and the anticipated benefit in progression-free survival comparing patients treated with an alemtuzumab-containing regimen is ≈6 months. Clearly, improved therapeutic strategies are needed.

## 5. Pathogenesis

Balanced chromosomal rearrangements involving T-cell receptor (TCR) enhancers on chromosome 14, occurring during thymocyte development [37], juxtapose one of three T-cell leukemia 1 family genes (most commonly TCL1A) with TCR, leading to aberrant, and pathognomonic, expression of TCL1A, TCL1B, or MTCP1 (mature T-cell proliferation) oncogenes in ≈95% of T-PLL [3]. While TCL1A overexpression is oncogenic in transgenic mice [18], deletions or inactivating mutations involving ataxia telangiectasia mutated (ATM) are also observed in >80% of T-PLL cases. CHEK2, a downstream ATM effector, is disrupted in a minority of cases [30]. The cooperativity observed between TCL1A overexpression and ATM loss is likely unique to T-PLL across the spectrum of T-cell lymphoproliferative neoplasms and, by cooperatively impairing genomic stability, fosters T-PLL leukemogenesis [19]. ATM orchestrates the DNA damage response and activates p53; notably, TP53 is deleted in only a minority of T-PLL cases [19,38]. Therefore, ATM loss substantially contributes to the complex karyotypes [19], and chemotherapy resistance observed in T-PLL [36]. Transcriptional profiling studies, while further highlighting the significance of recurrently altered pathways subject to recurrent copy number alterations (CNA) and single nucleotide variants (SNV; e.g., JAK/STAT pathway), provide further mechanistic insight (e.g., decreased expression of pro-apoptotic genes), explaining T-PLL resistance to cell death [19,39,40]. 

The hijacking of lineage-specific pathways, including those driven by antigen-, costimulatory-, and cytokine-receptor signaling, is a recurring motif across the spectrum of T-cell neoplasms [reviewed in [15]], and T-PLL is no exception. Expression of the TCR, and the downstream kinases associated with it (e.g., Lck, Zap-70), is observed in most T-PLL [35], thus implicating TCR signaling in disease pathogenesis. In fact, TCR engagement leads to the co-localization of TCL1A and phosphorylated AKT with constituents of the TCR signalosome, whereby TCL1A enhances basal and TCR-dependent AKT activation and proliferation [35,41]. More recently, genomic gains involving chromosome 8, which usually include c-Myc, have been shown to always involve the argonaute RISC catalytic component 2 (AGO2) locus [19]. AGO2 is classically associated with its endonuclease activity and role in miRNA/RNA silencing. However, AGO2 was recently shown to be associated with components of the TCR signalosome, including Zap-70, where it augmented TCR signaling [42]. Not surprisingly, then, TCR expression and activation (or AKT phosphorylation) are associated with inferior outcomes [35]. Transcriptional profiling also demonstrates that co-inhibitory (or “checkpoint”) receptors (e.g., CTLA-4, LAG-3, PD-1) are downregulated [19,41], thus providing further evidence for the TCR’s role in T-PLL pathogenesis, and potential utility as a therapeutic target. For example, IL-2 inducible T-cell kinase (ITK), a Tec family kinase and BTK homologue, plays a central role in TCR signaling and is an attractive therapeutic target in T-PLL [41,43], and other mature T-cell neoplasms [44,45]. 

Recurrent gain-of-function (GOF) mutations in JAK1, JAK3, and STAT5B are observed in T-PLL [19]. In a large meta-analysis, genomic alterations predicted to culminate in JAK/STAT signaling were observed in ≈90% of T-PLL cases, many of which were GOF JAK/STAT mutations [46]. These GOF alterations were also complemented by recurrent losses in phosphatases (e.g., DUSP4, SOCS3, CD45) that normally downregulate JAK/STAT signaling. Therefore, JAK/STAT pathway activation is a hallmark of T-PLL, and may cooperate with TCR-dependent signaling during disease pathogenesis, as elevated TCL1A expression was observed in JAK/STAT mutated T-PLL [46]. These findings may have therapeutic implications, as specific mutations may be associated with sensitivity (e.g., JAK) or resistance (e.g., STAT5B) to JAK inhibitors [47,48]. 

Despite the significant advances achieved over the past decade, the extent to which genetic or transcriptional alterations implicated in TCR-, costimulatory-, and JAK/STAT-mediated signaling in T-PLL are dependent upon, or supported by, constituents of the tumor microenvironment is poorly understood, and a potentially fruitful area for future studies.

## 6. Treatment

As there is no evidence to suggest that early treatment improves outcomes, asymptomatic patients may initially be observed [3]. However, most asymptomatic patients will develop active disease within 1–2 years of diagnosis [8,49]. The TPLL-ISG has suggested that disease-related fatigue, B symptoms, bone marrow failure (anemia, thrombocytopenia), symptomatic lymphadenopathy, hepatosplenomegaly, rapidly progressive lymphocytosis, or significant extranodal involvement (e.g., pleural effusion) represent symptomatic disease, and are thus indications for therapy [3]. 

### 6.1. Conventional Cytotoxic Agents

T-PLL is notoriously resistant to conventional cytotoxic chemotherapeutic agents; thus, responses are usually incomplete and rarely durable with this approach. In 15 T-PLL patients treated with cyclophosphamide, doxorubicin hydrochloride, vincristine sulfate, and prednisone (CHOP), there were five short-lived responses (including one complete response lasting three months) [6]. In one small series of patients treated with either CHOP or related regimens (e.g., chlorambucil/prednisone; cyclophosphamide, vincristine and prednisone), two out of twenty patients responded [7]. Alternative regimens, including pentostatin, bendamustine, and FMC (fludarabine, mitoxantrone, and cyclophosphamide), have been studied, but outcomes remain dismal, with an overall survival ≤9 months anticipated (Table 3). These findings demonstrate that T-PLL is a chemorefractory disease and are compatible with the increasing appreciation that its genetic landscape is associated with impaired DNA damage and apoptotic response.

SAMHD1, which has both dNTPase activity and dNTPase-independent functions regulating DNA replication, is recurrently mutated in T-PLL [19,57]. Given its dNTPase activity, SAMHD1 loss prevents the degradation of Ara-CTP, the active metabolite of cytarabine, and confers increased sensitivity to cytarabine in AML [58]. In T-PLL, SAMHD1 mutations were associated with decreased SAMHD1 expression and a corresponding increase in intracellular dATP, as anticipated [57]. However, a significant difference in cytotoxicity between wild-type and mutated SAMHD1 was not observed in T-PLL cells treated with cytarabine [58].

### 6.2. Alemtuzumab

Given the poor outcomes achieved with conventional chemotherapeutic agents, single-agent intravenous alemtuzumab has emerged as the standard of care in frontline settings for patients requiring treatment. The use of subcutaneous alemtuzumab appears to be inferior to intravenous alemtuzumab [50]. Multiagent treatment, including alemtuzumab, has not been shown to be superior to single-agent alemtuzumab in treatment-naïve patients [9]. For example, subcutaneous alemtuzumab combined with fludarabine, mitoxantrone, and cyclophosphamide followed by alemtuzumab maintenance did not produce results superior to those of single-agent intravenous alemtuzumab [59]. While alemtuzumab is associated with high response rates (summarized in Table 3), the responses achieved are not durable, and disease relapse is inevitable. Furthermore, prolonged lymphopenia after treatment discontinuation, viral reactivation, and other infectious complications remain a significant challenge with alemtuzumab [60,61]. Upon relapse, and depending upon the length of remission, alemtuzumab may be repeated; however, testing for CD52 expression is recommended as tumor cells may lose CD52 expression [62,63]. Repeating alemtuzumab has induced second and third remissions, but not surprisingly, these remissions are successively shorter [49]. In the case of leptomeningeal involvement, conventional intrathecal cytotoxic agents are often ineffective, and IV alemtuzumab has poor penetration of the CNS. Intrathecal alemtuzumab has previously demonstrated activity in a single case report and may be considered for refractory leptomeningeal disease [64]. 

### 6.3. Stem Cell Transplantation

Hematopoietic stem cell transplant (HSCT) is the only potentially curative therapy and should be considered in transplant-eligible patients upon achieving a response to cytoreductive treatment (usually alemtuzumab) (recently reviewed in [65]). Several studies demonstrate the benefit associated with allogeneic hematopoietic stem cell transplant (allo-HSCT), and are summarized in Table 4. However, it has not been shown that transplants increase survival. In the largest single-center series to date, for patients who achieved complete remission, HSCT was not associated with longer progression-free survival or overall survival [9]. Recently, the Center for International Blood and Marrow Transplant Research (CIBMTR) published a large study that included 266 patients with T-PLL who underwent allogeneic HSCT. Disease-free and overall survival rates at four years were 26% and 30%, respectively, and treatment-related mortality and relapse rates were 32% and 42%, respectively. Disease relapse was the most common (52%) cause of death in these patients [66]. If allogeneic transplantation is not possible, autologous HSCT can also be considered, as limited retrospective experience suggests that this approach may be associated with superior survival when compared to alemtuzumab alone [67] and may also lead to durable responses compatible with those observed in patients undergoing allogeneic HSCT [66]. 

## 7. Novel Therapies and Future Therapeutic Strategies

**Figure 4:**
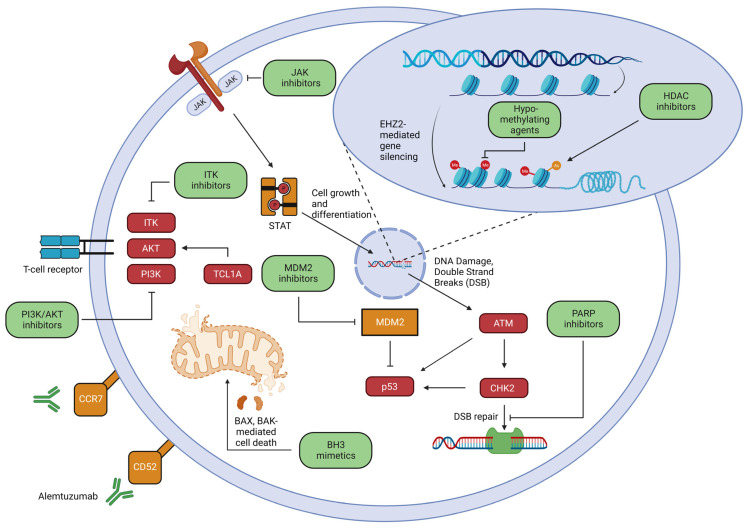
Therapeutic pathways in T-PLL. While alemtuzumab is the current frontline therapy, an improved understanding of T-PLL pathogenesis is leading to potentially targetable vulnerabilities. These include attempts to reinstate p53 function through MDM2 inhibitors as well as exploitation of faulty double strand break repair via PARP inhibitors, inhibition of TCR activation through ITK inhibitors as well as AKT/PI3K inhibitors, JAK inhibitors which inhibit cell growth and differentiation through the JAK-STAT pathway, BH3 mimetics to promote BAX and BAK mediated cell death, epigenetic approaches including hypomethylating agents and HDAC inhibitors, and novel antibody targeting e.g., CCR7. The illustration was created using Biorender.com and is adapted from previous review articles [47,72,73].

Improved understanding of T-PLL pathogenesis has exposed novel vulnerabilities that are currently being explored pre-clinically and in early-phase clinical trials (Table 5), some of which have been recently reviewed [72,73], and have been identified in an unbiased ex vivo drug screen [47]. These novel therapeutic strategies (represented in Figure 4), informed by an improved understanding of T-PLL pathogenesis, suggest that we may be on the dawn of a new era in T-PLL management.

### 7.1. MDM2 Inhibition

ATM, CHK2, and/or p53 are functionally impaired in T-PLL. Given their role in DNA repair, cell cycle progression, and the induction of apoptosis (Figure 4), these aberrancies likely contribute to the chemorefractory phenotype observed in T-PLL. Notably, p53 is not mutated or deleted in most T-PLL, but is functionally impaired due to upstream ATM and/or CHEK2 loss. Therefore, the inhibition of MDM2, a p53 ubiquitin ligase, is a potentially promising strategy. An MDM2 inhibitor, idasanutlin, increased p53 activity in T-PLL cells, culminating in apoptosis, and this effect was more pronounced with the addition of an HDAC inhibitor, Panobinostat [19]. These findings are compatible with those observed in other p53-proficient T-cell lymphomas [82]. Clinical trials investigating MDM2 inhibition are ongoing (Table 5). The impaired DNA double strand break repair observed in ATM-deficient cells may confer sensitivity to PARP inhibition, and has been observed in ATM-deficient CLL [75]. Olaparib was tested in a phase I trial that included two T-PLL patients, both with mutated ATM, and one transiently responded [83]. 

### 7.2. TCR Signaling

TCR expression is observed in most T-PLL cases and is intimately involved in pathogenesis; therefore, the inhibition of TCR-driven kinases warrants study. Herling et al. investigated TCR-dependent PI3K/AKT activation and demonstrated that pharmacologic PI3K/AKT inhibitors reduced growth of T-PLL cells [35], whereas only a subset of T-PLL patients responded to PI3K/AKT inhibition in another study [47]. Considering the success of Bruton’s tyrosine kinase inhibitors in B-cell malignancies, there has been interest in ITK inhibition. ITK has an analogous role in TCR signaling [44], but also has a redundant role with resting lymphocyte kinase (RLK), another member of the TEC family of kinases [45,84]. A reversible ITK/RLK inhibitor (PRN694) has been shown to impair both TCR signaling and T-PLL expansion following TCR engagement [43].

### 7.3. JAK/STAT Inhibition

Gain-of-function mutations involving JAK/STAT family members, particularly JAK1, JAK3, and STAT5B, are recurrent in T-PLL, thus providing a rationale for JAK inhibition [19,47,85]. In an unbiased screen performed in a well-characterized cohort of T-PLL specimens, ruxolitinib was among the top 50 most active novel agents [47]. While JAK1/JAK3 mutations were associated with sensitivity to ruxolitinib, a particular STAT5B mutation (N642H, located within the STAT5B SH2 domain) was associated with resistance. STAT5B N642H, described in other T-cell neoplasms [85,86], is located on the SH2 binding site [86], and is a gain-of-function mutation [19,86], likely explaining its association with ruxolitinib resistance. Interestingly, STAT5B Y665F, which is also a gain-of-function mutation [85,86], was associated with ruxolitinib sensitivity. Whether this may be explained by the inhibition of alternative STATs is unclear, but certainly feasible given the recurrent losses of negative regulatory phosphatases (e.g., DUSP4, SOCS3) in T-PLL [19]. Consistent with these findings, Gomez-Arteaga et al. treated a patient with relapsed JAK3 mutated T-PLL with a combination of both ruxolitinib and tofacitinib, as pre-clinical ex vivo studies using the patient’s T-PLL cells demonstrated an additive effect with the combination [74]. While a clinical response was not achieved with treatment, disease stabilization and modest improvements in the patient’s absolute lymphocyte count (not satisfying criteria for a partial response) and regression of leukemia cutis were observed over 10–11 months of treatment. By comparison, the time to the next treatment for the two prior lines of therapy (e.g., romidepsin/lenalidomde; alemtuzumab) was 5–8 months. It should be noted that ruxolitinib sensitivity is unlikely to be limited to JAK/STAT mutated cases, as many non-mutated cases are also sensitive [47], thus highlighting the potential importance of JAK/STAT signaling in T-PLL generally. In addition, most JAK/STAT mutations are subclonal [19]. Therefore, ruxolitinib, or other JAK inhibitors may be rationally combined with other novel agents. For example, a large BH3 profiling study demonstrated that T-PLL cells are variously dependent on Bcl-1 or Mcl-1 [78]. However, pre-treatment with either belinostat or ruxolitinib significantly increased Bcl-2 dependence, thus suggesting that venetoclax may be rationally combined with these agents in T-PLL; strong synergy with these combinations was observed in JAK/STAT mutated, but not wild-type, cases in ex vivo studies. These authors went on to treat a JAK3 mutated patient with the combination of ruxolitinib and venetoclax, and a partial (but near complete) response was achieved and ongoing at 10 months [78]. A patient without an identified JAK/STAT mutation was similarly treated but after disease progression on single-agent venetoclax. The addition of ruxolitinib led to disease stabilization. Informed by these findings, 15 patients with relapsed/refractory T-PLL were treated with ruxolitinib and venetoclax [79]. JAK/STAT pathway mutations were observed in most patients (n = 12). The overall response rate was 73%, and five responses were nearly complete, all in the mutated group. In contrast, one of three patients in the JAK/STAT wild-type group responded. A phase I clinical trial (NCT03989466) of alemtuzumab and itacitinib (a JAK1 inhibitor) for the treatment of both treatment-naïve and relapsed/refractory T-PLL is currently recruiting patients. 

### 7.4. BH3 Mimetics 

As noted, T-PLL cells are variously dependent on Bcl-2 and Mcl-1, both of which may be regulated by JAK/STAT and TCR signaling [78,87]. In a large high-throughput screen using primary specimens ex vivo [76], T-PLL was observed to be particularly sensitive to venetoclax, consistent with subsequent studies [76], and in vitro sensitivity predicted a clinical response in two T-PLL patients treated with venetoclax. The first patient treated achieved a >80% reduction in leukemic burden 12 hours after the first dose of venetoclax, and the second patient achieved a partial response. Hampel et al. reported outcomes in a small cohort of T-PLL patients variously treated with venetoclax alone or in combination with bendamustine [80]. Among the four patients treated with single-agent venetoclax, one patient achieved disease stabilization. Among the five patients treated with venetoclax and bendamustine, the overall response rate (all partial) was 80%. Ibrutinib, which inhibits ITK pre-clinically [44], but is associated with suboptimal ITK occupancy (≈50%) clinically [88], showed promising ex vivo synergy in combination with venetoclax [77]. However, a clinical trial testing this combination enrolled 14 patients with a single observed response and reported progression-free survival of less than 3 months (NCT03873493). 

Efficient RNA polymerase II transcription, including oncogenic loci, is regulated by the cyclin-dependent kinase CDK9 [89,90]. Therefore, CDK9 inhibition leads to transcriptional “pausing” and inefficient gene transcription. In preliminary studies in T-PLL, CDK9 inhibition decreased the expression of JAK/STAT related transcripts, c-Myc, and Mcl-1 [as has been observed in other hematologic malignancies [91,92]]. Given the increased expression of c-Myc in T-PLL [19], and the role of CDK9 in Myc-addicted cancers [93], in conjunction with the Mcl-1 dependence observed in a subset of T-PLL [80], CDK9 inhibition is an attractive and highly rational approach. Not surprisingly, a novel CDK9 inhibitor induced apoptosis in primary T-PLL cells and was also synergistic with venetoclax [91]. As CDK9 inhibitors are now in early-phase clinical trials, future studies in T-PLL are warranted.

### 7.5. Epigenetic Approaches 

Aberrant DNA repair and histone modification mechanisms are recurrently observed in T-PLL, yet p53 remains functionally intact in most T-PLL and is positively regulated by post-translational acetylation [94], suggesting that histone deacetylase inhibitors (HDACi) may be rationally combined with either conventional [81] or novel agents in T-PLL, including MDM2 inhibitors [19] and venetoclax [80]. Of course, most HDACi in clinical use, including those used in these studies, are non-selective. Interestingly, a comparison of HDAC expression between T-PLL and normal T-cells demonstrated a significant increase in HDAC6 expression in T-PLL, whereas no difference in expression was observed for the other HDAC’s examined [95]. Furthermore, the increased HDAC6 expression was selectively observed in T-PLL as a similar increase in HDAC6 was not observed in the other T-cell neoplasms (e.g., PTCL, NOS; AITL; ALCL; T-ALL) examined. More importantly, a selective HDAC6 inhibitor (KT-531) impaired the viability of T-PLL cells, and in contrast to pan-HDACi, had little to no activity in other T-cell neoplasms. Consistent with other studies, KT-531 also demonstrated synergy with both an MDM2 inhibitor and venetoclax.

EZH2, a member of the polycomb repressive complex 2 (PRC2) that trimethylates H3K27, is a transcriptional repressor and is recurrently mutated in T-PLL [19,96]. These gain-of-function mutations observed in other B- and T-cell neoplasms confer increased sensitivity to EZH2 inhibition and warrant further study in T-PLL. Interestingly, EZH2 phosphorylation by JAK3 functionally transforms EZH2, converting it to a transcriptional coactivator that promotes the upregulation of genes involved in cell proliferation [97]. To the best of our knowledge, the extent to which EZH2 is a transcriptional repressor or activator, and the extent to which this may be associated with JAK3 mutational status, has not yet been explored in T-PLL, but has therapeutic implications.

### 7.6. Monoclonal Antibodies: Moving beyond Alemtuzumab

While alemtuzumab is associated with high response rates, these responses are not durable, and are frequently associated with significant infectious complications. However, the experience with alemtuzumab suggests that monoclonal antibody-based approaches against alternative targets are attractive, particularly if depletion of normal cells expressing these targets is associated with less significant immunosuppression. With this in mind, it is notable that the chemokine receptor CCR7 is highly expressed by T-PLL cells [98]. Ligand binding (CCL19, CCL21) was associated with increased CCR7-dependent proliferation of T-PLL cells, and CCR7 expression itself was associated with inferior overall survival [98]. A CCR7 monoclonal antibody, by blocking ligand-dependent CCR7 signaling, impaired T-PLL migration and proliferation but also increased cell-mediated cytotoxicity in ex vivo and xenograft models, thus highlighting the potential utility of this strategy [98]. 

## 8. Conclusions

T-PLL remains a rare and largely incurable disease, and the outcomes remain dismal. However, an improved understanding of T-PLL pathogenesis and its genetic landscape have unveiled novel therapeutic targets. Future studies investigating these novel therapeutic strategies are needed, and in keeping with the recently performed pre-clinical studies highlighted here, will increasingly be informed by an improved understanding of this disease, suggesting that we may be on the dawn of a new era in T-PLL therapeutics. Therefore, clinical trial participation should be encouraged.

## Figures and Tables

**Table 1:** Mature T-cell lymphoproliferative disorders with a leukemic presentation.

	T-PLL [6,7,11,18,19]	T-LGL [20,21,22]	ATLL [16,23,24]	NK-Cell LGL [21]	Sézary Syndrome [25,26,27]	Aggressive NK-Cell Leukemia [17,28]
Epidemiology	The incidence in Western countries is 2 per 1,000,000 people per year. T-PLL accounts for about 2% of mature leukemias.	Accounts for 2% to 5% of chronic lymphoproliferative disorders. The overall incidence is 0.72 per 1,000,000 people per year.	More common in parts of the world that are endemic for HTLV-1, including Japan, the Caribbean, and Central and South America. The global estimation of new cases is approximately 3000 per year.	Accounts for less than 5% of LGL disorders, is predominantly observed in Asia, and tends to affect individuals of younger age.	The annual incidence rate of SS is 1 per 10 million per year. SS represents 3% of all cutaneous lymphomas.	A very rare disease, predominantly in Asia. Median age 37 years (range 10–78). More frequent in males than females (male-to-female ratio: 1.3:1).
White Blood Cell Count (WBC)	Most patients present with extreme lymphoctosis (≥100,000/µL).	1.6–49.1 × 10^9^/L (median count 5.5 × 10^9^/L)	Acute type: 3.9–296 × 10^9^/L (median count 88 × 10^9^/L). Chronic type: 10.4–17 × 10^9^/L (median count 17 × 10^9^/L). Lymphomatous type: 1.1–45.3 × 10^9^/L (median count 7.5 × 10^9^/L).	Similar WBCs as in T-LGL. Blood LGL count of >700/μL.	The median white blood cell count is 21 × 10^9^/L. The Sézary cell count is ≥1000 cells/μL.	WBC count in aggressive NK-cell leukemia varies widely and can be high, with a median count of 2.80 × 10^9^/L (range, 0.29–57 × 10^9^/L).
Histology	Three morphologic variants have been recognized (see Section 3).	Neoplastic cells cannot be differentiated cytologically from normal reactive cytotoxic lymphocytes.	In the acute type, neoplastic cells feature hyperchromatic nuclear chromatin and deeply lobulated nuclear contours: “flower cells”. In the chronic type, the neoplastic cells show subtle cytological atypia.	The histological features of NK-cell LGL are indistinguishable from those of T-LGL.	Atypical lymphocytes with cerebriform nuclei that are larger than normal lymphocytes and have hyperchromatic nuclei. These features are not specific, as similar morphological features can be observed in reactive lymphocytes in autoimmune disorders.	Neoplastic cells are medium to large, with moderate amounts of basophilic agranular cytoplasm, highly irregular nuclear contours, and a distinct open chromatin pattern with visible nucleoli.
Phenotype	Usually CD3+. CD4+ and CD8− is most common, however co-expression of CD4 and CD8 is also observed.	CD3+, CD16+, CD27−, CD28−, CD45R0−, CD45RA+, and CD57+. Ta/b > Tg/d. CD8+ > CD4+. CD94 is positive in 20% of CD8+ and in 2% of CD4+ cases.	Preserved expression of pan-T-cell markers (CD2, CD3, CD4, CD5) with strong expression of CD25 and FoxP3, and frequent loss of CD7.	Characterized by CD2+, sCD3−, CD3e+, TCRa/b−, CD4−, CD8+, CD16+, and CD56+. Expression of CD94 is detected in more than 95% of the cases. KIR expression is detected in 53% of cases.	Usually CD3+, CD4+ and CD8−. The aberrant loss of pan-T-cell antigens, including CD2, CD3, CD4, CD5, CD7, and/or CD26 is frequently seen.	Characterized by the expression of CD2, cytoplasmic CD3, CD7, CD16, and CD56, while the expression of surface CD3 is negative. Expression of CD94 is detected in more than 95% of cases.
Molecular features	TCL1A overexpression, combined with loss of ATM appears unique to T-PLL (see Section 5).	Activating mutations in genes such as *STAT3*-*STAT5b*, and *NFKB* are common.	Most cases exhibit intricate cytogenetic abnormalities that result from the detrimental impact of the viral-encoded TAX. Recurrent loss-of-function mutations in CIC/ATXN1 complex are detected in up to 50% of cases.	Mutations in *STAT3* are observed in less than 10% of the cases, and *STAT5b* mutations are not detected.	Commonly altered genes in Sézary syndrome include *ZEB1*, *STAT5B*, *FAS*, *ARID1A*, *CDKN2A*, and *TP53*.	Activating mutations in *JAK-STAT* pathway genes, particularly *STAT3* and *STAT5B* are detected. Mutations in tumor suppressor genes, such as *TP53* are frequent.
Clinical features	Generally presents with extreme lymphocytosis, anemia and thrombocytopenia in the 6th to 7th decade of life.	At the time of diagnosis, approximately one-third of patients are asymptomatic. The initial presentation is typically associated with cytopenias (anemia or neutropenia).	Caused by HTLV-1 infection. The acute and chronic types usually present with leukemia, hepatosplenomegaly, and hypercalcemia. The lymphomatous type does not exhibit leukocytosis or hepatosplenomegaly.	Characterized by a chronic and indolent clinical course, that is similar to T-LGL. The 10-year survival rate is around 70%.	Characterized by erythroderma with severe pruritus and generalized lymphadenopathy.	Patients typically present with fever, hepatosplenomegaly, hemophagocytic lymphohistiocytosis and hyperferritinemia.

**Table 2:** Major and minor diagnostic criteria for T-PLL defined by TPLL-ISG *.

Major Criteria
1. >5 × 10^9^/L cells of T-PLL phenotype in peripheral blood or bone marrow
2. T-cell clonality (by polymerase chain reaction for T-cell receptor beta or gamma, or by flow cytometry) 3. Abnormalities of 14q32 or expression of *TCL1A/B* or *MTCP1*
**Minor Criteria**
1. Abnormalities of chromosome 11 (11q22.3, *ATM*)
2. Abnormalities of chromosome 8 [idic(8)(p11), t(8;8), or trisomy 8(q)] chromosome 5, 12, 13, or 22
3. Complex karyotype
4. Involvement of T-PLL specific site (e.g., splenomegaly or pleural effusions)

* Adapted from [3].

**Table 3:** Summary of overall response rate (ORR), complete response (CR), median progression-free survival (PFS) and median overall survival (OS) by regimen.

References	Regimen	Disease Status	n	ORR	CR	Median PFS (Months)	Median OS (Months)
[9,50,51,52]	IV alemtuzumab **	Frontline	80	85	71	11	15
		Salvage	183	57	50	5	11.3
[50]	Subcutaneous alemtuzumab	Frontline	9	33	33	-	-
[53]	Bendamustine	Frontline	6	67	33	2	7
		Salvage	9	44	11	0	3
[9,54]	IV alemtuzumab and pentostatin **	Frontline	13	82	73	4.3	10.4
		Salvage	18	70	59	5.2	6.4
[55]	IV alemtuzumab and FMC	Frontline	16	92	48	11.5	17.1
		Salvage	9				
[56]	Pentostatin	Salvage	55	45	9	6	9

** Composite of multiple trials, weighted by n.

**Table 4:** Summary of trials of T-PLL with allo-HSCT ***.

Reference	n	CR (%) at allo-HSCT	PR (%) at allo-HSCT	OS	Relapse Rate
[68]	37	60%	27%	4-year OS: 42%	38% at 4 years
[69]	27	51%	37%	3-year OS: 36%	47% at 3 years
[70]	11	81%	9%	4-year OS: 56%	21% at 4 years
[71]	20	30%	5%	3-year OS: 39.8%	69.6% at 3 years
[66]	266	56%	30%	4-year OS: 30%	41.9% at 4 years

*** Adapted from [66].

**Table 5:** Summary of clinical investigations of novel therapies.

Reference	Regimen	Disease Status	Study Type	n	Result
[74]	Tofacitinib and ruxolitinib	Salvage	Case Report	1	Patient had 10 months of partial response followed by eventual progression
[75]	Olaparib	Salvage	Phase I clinical trial	2	Patient A: treated for 133 days w/disease progression. Patient B withdrew after 14 days given dose limiting toxicity
[76]	Venetoclax	Salvage	Case Report	2	Patient A: partial response however died with fulminant sepsis 18 days after treatment initiation. Patient B: partial response, at 131 days on venetoclax, disease relapse
[77]	Venetoclax and ibrutinib	Salvage	Case Report	2	Patient A: treated for 40 days. Course was complicated by influenza A infection and patient transitioned to comfort care. Patient B: treated for 80 days; however, patient died of bacterial pneumonia.
[78]	Venetoclax and ruxolitinib	Salvage	Case Report	2	Patient A: 10 months of partial response. Patient B: progression on day 127 following therapy, then lost to follow-up.
[78]	Venetoclax and romidepsin	Salvage	Case Report	1	Partial response ongoing, 9 months, at time of publication of case.
[79]	Venetoclax and ruxolitinib	Salvage (n = 14) Frontline (n = 1)	Multicenter retrospective study	15	ORR 73% (all were partial response), median PFS 1.8 months in wild-type JAK/STAT vs. 5.6 months in mutated JAK/STAT pathway ‡
[80]	Venetoclax; 5 patients received bendamustine	Salvage	Single-center retrospective study	9	Disease control rate 56% (best response was PR in 4 and stable disease in 1)
[81]	Alemtuzumab and cladribine, some received vorinostat, romidepsin, and brentuximab	Salvage (n = 6) Frontline (n = 2)	Single-center prospective study	8	ORR 100% (88% CR, 12% PR), median OS 25.7 months, 38% were alive at time of publication.
NCT03873493	Venetoclax and Ibrutinib	Salvage	Phase II Clinical Trial	14	ORR 7.1%, median PFS 2.7 months
NCT01186640	FMC-subcutaneous alemtuzumab induction followed by alemtuzumab	Salvage (n = 4) Frontline (n = 12)	Phase II Clinical Trial	16	ORR 68.8% (31.3% CR, 37.5% PR), median OS 16.7 months, median PFS 11.2 months
NCT04496349	APG-115 (MDM2 inhibitor) +/− APG-2575 (Bcl-2 inhibitor)	Salvage	Phase II Clinical Trial	36 †	Recruiting; estimated study completion date: 31 May 2024
NCT03989466	Alemtuzumab and Itacitinib (JAK1 inhibitor)	Frontline or salvage	Phase I Clinical Trial	15 †	Recruiting; estimated study completion date: 21 December 2023

‡ *p* < 0.05; † estimated enrollment.

## Data Availability

No new data were created or analyzed in this study. Data sharing is not applicable to this article.
